# Lack of Evidence on Association between Iron Deficiency and COVID-19 Vaccine-Induced Neutralizing Humoral Immunity

**DOI:** 10.3390/vaccines11020327

**Published:** 2023-02-01

**Authors:** Arwa A. Faizo, Asma A. Bawazir, Majed N. Almashjary, Ahmed M. Hassan, Fadi S. Qashqari, Ahmed S. Barefah, Sherif A. El-Kafrawy, Thamir A. Alandijany, Esam I. Azhar

**Affiliations:** 1 Special Infectious Agents Unit, King Fahd Medical Research Center, King Abdulaziz University, Jeddah 21589, Saudi Arabia; 2 Department of Medical Laboratory Sciences, Faculty of Applied Medical Sciences, King Abdulaziz University, Jeddah 21589, Saudi Arabia; 3 Hematology Research Unit, King Fahd Medical Research Center, King Abdulaziz University, Jeddah 21589, Saudi Arabia; 4 Department of Microbiology, College of Medicine, Umm Al-Qura University, Makkah 24381, Saudi Arabia; 5 Department of Hematology, Faculty of Medicine, King Abdulaziz University, Jeddah 21589, Saudi Arabia

**Keywords:** COVID-19 vaccine, iron deficiency, SARS-CoV-2, humoral immunity, ELISA

## Abstract

Iron is a crucial micronutrient for immunity induction in response to infections and vaccinations. This study aimed to investigate the effect of iron deficiency on COVID-19-vaccine-induced humoral immunity. We investigated the effectiveness of COVID-19 vaccines (BNT162b2, mRNA-1273, and ChAdOx nCov-2019) in iron-deficient individuals (*n* = 63) and provide a side-by-side comparison to healthy controls (*n* = 67). The presence of anti-SARS-CoV-2 spike (S) and anti-nucleocapsid (NP) IgG were assessed using in-house S- and NP-based ELISA followed by serum neutralization test (SNT). High concordance between S-based ELISA and SNT results was observed. The prevalence of neutralizing antibodies was 95.24% (60/63) in the study group and 95.52% (64/67) in the controls with no significant difference. The presence/absence of past infection, period since vaccination, vaccine type, and being iron-deficient or having iron-deficiency anemia did not exert any significant effect on the prevalence or titer of anti-SARS-CoV-2 neutralizing antibodies. NP-based ELISA identified individuals unaware of exposure to SARS-CoV-2. Moreover, absence of anti-NP IgG was noted in participants who were previously diagnosed with COVID-19 suggesting the unpredictability of after-infection immunity. To sum up, this study demonstrated an initial lack of evidence on the association between iron deficiency and the effectiveness of COVID-19-vaccine-induced neutralizing humoral immunity. Similar studies with larger sample size remain necessary to obtain comprehensive conclusions about the effect or lack of effect of iron on COVID-19-vaccine effectiveness.

## 1. Introduction

Iron deficiency (ID) is a global health problem that affects nearly two billion people [1], and iron deficiency anemia (IDA) is considered the topmost leading cause of anemia worldwide [2]. The prevalence of ID and IDA is generally higher in women of child-bearing age, preschool children, and individuals with low socioeconomic status [1,3]. The underdeveloped regions have a five-time higher prevalence rates of ID and IDA compared to the developed nations [4]. This detrimental situation might have been worsened in recent years due to COVID-19 impact on food security [5].

Iron is an important nutrient for the development of efficient immune response to infections and vaccinations [6,7,8,9]. The mechanisms underlying reduced effectiveness of host immunity under iron-deprived state is not fully understood. However, iron is required for monocyte/macrophage differentiation and antimicrobial activity (e.g., the nicotinamide adenine dinucleotide phosphate hydrogen(NADPH)-dependent oxidative burst) [10]. Iron is also necessary for lymphocyte proliferation and differentiation as well as cytokines production [11,12]. Under the iron-deprived state, the immune system undergoes different alterations such as reduction in the number of CD4+ and CD8+ lymphocytes, and the levels of inflammatory cytokines [10,11,12]. Hypoferremia in animal models substantially decreased the effectiveness of immune response mediated by the effector and memory cells following vaccinations such as tetanus toxoid and *Mycoplasma hyopneumoniae* [7,13]. An independent study demonstrated suboptimal protective immunity induction following receiving diphtheria, pertussis, and pneumococcal (DPT) vaccines in children with ID compared to those with normal iron levels [14]. On the other hand, simultaneous administration of iron-fortified micronutrient powders with measles vaccine enhanced the antibody avidity and seroconversion of the vaccine recipients [14]. Another investigation conducted on humans found that some individuals possess defective B- and T-cells due to a rare mutation that interferes with cellular iron uptake [15].

In COVID-19 patients, hypoferremia and low transferrin saturation were marked as predictors for severe illness (e.g., hospitalization and admission to intensive care unit) [16,17,18,19,20]. It is not entirely clear how hypoferremia may worsen the outcome of COVID-19. Inefficient cellular oxygen sensing, impaired response to hypoxia, and reduction of lymphocyte count and activities are potential mechanisms underlining severe COVID-19 illness in individuals with hypoferremia [16,17,18,19,20]. With the introduction of COVID-19 vaccines, studies on vaccine safety and effectiveness are required to assess the actual level of protection afforded by immunization [21,22]. One of the recommendations raised by experts is to "correct the iron deficiency before administration of the COVID-19 vaccine" [23]. This is largely because of previous reports highlighting the necessity of iron for host immunity. However, there is lack of sufficient evidence about COVID-19 vaccine effectiveness per se in vaccine recipients with ID or IDA profile. Hence, we aimed in this study to investigate the effectiveness of COVID-19 vaccines in inducing protective humoral immunity in ID and IDA individuals and provided a direct side-by-side comparison with healthy controls.

## 2. Materials and Methods

### 2.1. Study Population and Demographic Data

The study was approved by the Biomedical Research Ethics Committee of Umm Al Qura University (protocol code HAPO-02-K-012-2021-09-747 and date of approval 7 September 2021). We invited students and staff at the College of Medicine, Umm Al-Qura University in Makkah and the faculty of applied medical sciences, King Abdulziz University in Jeddah through announcements and personal invitations to eligible candidates. Informed consent was obtained from all subjects involved in the study. A total number of 130 (65 female and 65 male) participants were enrolled in the study. The subjects’ demographic data were obtained through questions provided along with the consent forms. The demographic data included age, gender, and comorbidities, and awareness and dates of previous COVID-19 infections, as well as the COVID-19 vaccine profile. All participants had had double shots (either homologous or heterologous) of COVID-19 vaccines belonging to BNT162b2 by Pfizer-BioNTech, mRNA-1273 by Moderna, or ChAdOx nCov-2019 by AstraZeneca.

### 2.2. Hematological and Biochemistry Testing

Venous blood samples were drawn from all participants in plain tubes and ethylenediamine tetra-acetic acid dipotassium salt (EDTA-2K) tubes. Plain tubes were centrifuged at 3500 rpm for 5 min, after which ferritin level was determined by the Alinity system (Abbott Laboratories, IL, USA) that utilizes chemiluminescent microparticle immunoassay (CMIA). The EDTA-2K tubes were used to assess the hemoglobin level through complete blood count (CBC) utilizing the modern automated hematology analyzer (Sysmex Corporation, Kobe, Japan).

### 2.3. Serological Assays

Sera were subsequently subjected to in-house immunoassays: S-based and NP-based indirect ELISA, and serum-neutralization (SN) assay to assess the presence and activity of anti-SARS-CoV-2 antibodies. The detailed protocols for these immunoassays were previously described. The cut-off optical density values for S-based and NP at 450 nm were 0.27 and 0.17, respectively, while SN titers of ≥1:20 were considered positive. Our local clinical isolate of SARS-CoV-2 (SARS-CoV-2/human/SAU/85791C/2020, gene bank accession number: MT630432) was utilized in SN assay.

### 2.4. Statistical Analysis

All statistical analysis and graphing was done using GraphPad prism 9 software (GraphPad Software, La Jolla, CA, USA). Mann–Whitney U test, Kruskal–Wallis test and Fisher’s exact test were done as appropriate with *p* value of ≤0.05 considered statistically significant.

## 3. Results

**Figure 1:**
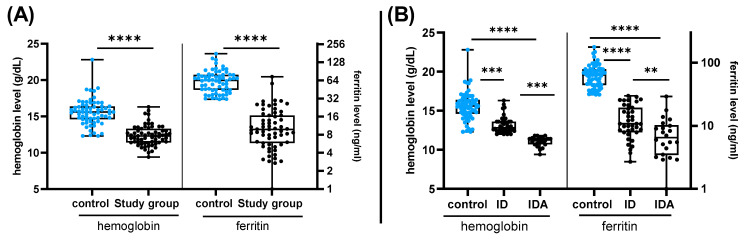
The levels of hemoglobin and ferritin among control and study group. (**A**) Hemoglobin levels (g/dL) and ferritin levels (ng/mL) for control group and study group (ID and IDA combined). (**B**) Hemoglobin and ferritin levels for control group and study group (separated as ID and IDA). Boxes represents 25th to 75th percentile range, black line demonstrates the median, and whiskers show minimum and maximum values. p values were calculated by Mann–Whitney U test and Kruskal–Wallis test, as appropriate. ** = *p* value < 0.01, ***, = *p* value < 0.001, **** = *p* value < 0.0001.

A total number of 130 participants were recruited in this study. The participants belonged to both genders, with a mean age of 21.9 years. All participants had received two homologous or heterologous doses of COVID-19 vaccines belonging to BNT162b2 by Pfizer-BioNTech, mRNA-1273 by Moderna, or ChAdOx nCov-2019 by AstraZeneca. The participants were divided into a control group and a study group based on their hemoglobin and ferritin levels, where a hemoglobin level of less than 13 g/dL for men and 12 g/dL for women coinciding with a ferritin level below 30 ng/mL indicated IDA, while a normal hemoglobin occurring with a ferritin level below 30 was considered as an insufficient iron store or iron ID. The cut-off of ferritin < 30 ng/mL was used because it was shown to exhibit high sensitivity and specificity (92% and 98%, respectively), to diagnose ID [24,25,26]. ID (*n* = 41) and IDA (*n* = 22) together comprised the entire study group (*n* = 63), while the control group comprised all other subjects with normal hemoglobin and ferritin levels (*n* = 67). Figure 1 shows the difference between the hemoglobin and ferritin levels for the controls and study group. The characteristics of the control and study groups are summarized in Table 1. All data shown on the table were extracted from the questionnaire provided to the participants at the time of recruitment with the exception of hemoglobin and ferritin levels that were determined as described in the Materials and Methods section. Statistical analysis revealed no significant difference between the control and study groups in most variables including age, BMI, previous diagnosis with COVID-19, and vaccination profile. On the other hand, hemoglobin and ferritin levels were significantly lower in the study group compared to the controls (Table 1 and Figure 1A). This remained true whether data of ID and IDA individuals were combined or separated with further significant reduction detected among IDA participants (Figure 1B).

**Figure 2:**
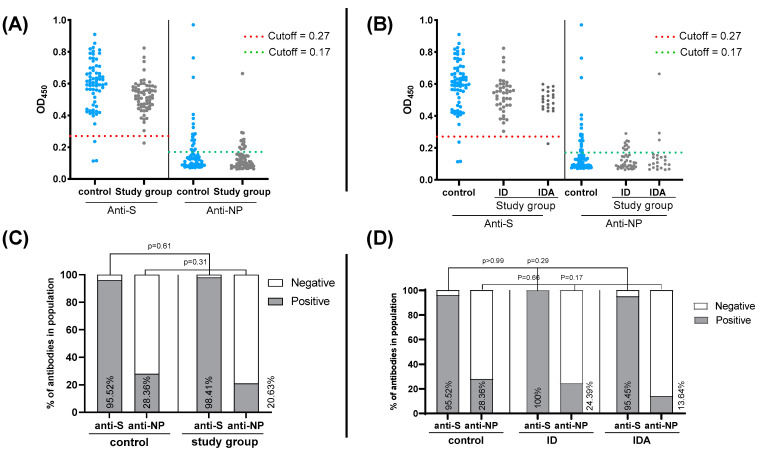
Optical density (OD) values and the prevalence of anti- SARS-CoV-2 S and NP IgGs among control group (normal hemoglobin and ferritin) and study group (ID, IDA). (**A**,**B**) Optical density values at 450 nm (OD450) as obtained from S- and NP-based ELISAs for control and study group: (**A**) ID and IDA combined and (**B**) ID and IDA separated. Dashed red lines represent the cut-off value for S-ELISA (OD450 = 0.27) and dashed green lines represent NP-ELISA cut-off value (OD450 = 0.17). (**C**,**D**) The prevalence (%) of anti-S and anti-NP in control and study group: (**C**) ID and IDA combined and (**D**) ID and IDA separated. *p* values were calculated by Fisher’s exact test, *p* value < 0.05 is considered significant.

To investigate COVID-19-vaccine effectiveness in producing anti-S IgGs, an S-based in-house ELISA was performed for all samples. In all 130 samples, anti-S IgG was identified in 126 (96.92%) samples with OD_450_ values > 0.27. The prevalence rates of anti-S IgG for the study and control groups were 98.41% (62/63) and 95.52% (64/67), respectively, with no significant difference observed (*p* = 0.61, 95% CI = 0.02612 to 2.377). To exclude any possible interference caused by previous COVID-19 infections, an NP-based in-house ELISA was performed because all types of vaccines received by the participants specifically mount anti-S antibodies. Anti-NP IgG was detected in 32/130 sample (24.62%) with OD_450_ values > 0.17. The prevalence rate of anti-NP IgG for the study group was 20.63% (13/63) and for the control was 28.36% (19/67), showing non-significant difference (*p* = 0.31, 95% CI = 0.6609 to 3.467). Our findings remained consistent whether data from ID and IDA participants were combined or separated (Figure 2 right and left panels, respectively).

**Figure 3:**
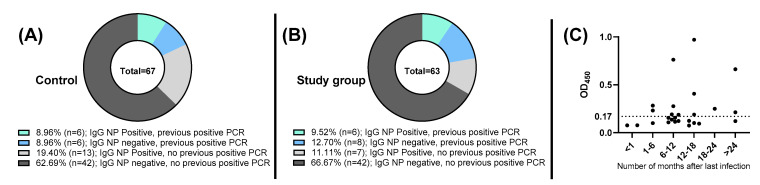
Analysis of anti-SARS-CoV-2 NP IgG in control group (normal hemoglobin and ferritin) and study group (ID, IDA). (**A**) Prevalence of IgG NP among control group. (**B**) Prevalence of IgG NP among study group. (**C**) OD450 readings of NP-based ELISA across different time periods among participants who had reported previous positive COVID-19 PCR result in the entire population. Dashed black line represents NP-based ELISA cut-off (OD450 = 0.17).

Further analysis performed on IgG NP findings revealed that 62.50% (20/32) did not belong to participants who reported previous COVID-19 infection but rather, belonged to those who did not report any previous infection (Figure 3A,B). Interestingly, 53.83% (14/26) of those who reported a previous infection tested negative for IgG NP. These findings indicate that many participants with positive IgG NP were unaware of a possible COVID-19 infection acquisition prior the time of the study. In addition, the noticed variability with IgG NP results despite the time after infection suggests the unpredictability of after-infection immunity (Figure 3C).

**Figure 4:**
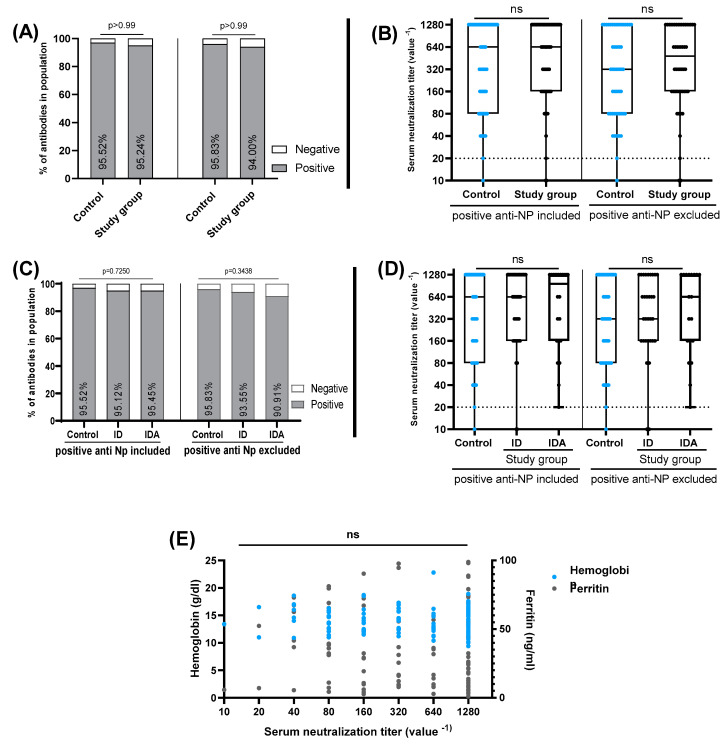
The prevalence of COVID-19 neutralizing antibodies in the control group (normal hemoglobin and ferritin) and study group (ID, IDA). (**A**) The prevalence (%) of neutralizing antibodies in control group and study group (ID and IDA combined) when anti-NP sero-positive cases were included (left) and excluded (right). *p* values were calculated by Fisher’s exact test. (**B**) Comparison of neutralizing antibody titers among control group and study group (ID and IDA combined) when anti-NP sero-positive cases were included (left) and excluded (right). (**C**) The prevalence (%) of neutralizing antibodies in control group and study group (ID and IDA separated) when anti-NP sero-positive cases were included (left) and excluded (right). *p* values were calculated by Fisher’s exact test. (**D**) Comparison of neutralizing antibody titers among control group and study group (separated as ID and IDA) when anti-NP sero-positive cases were included (left) and excluded (right). Dashed black line indicates the cut-off value (SN titer ≥ 1:20 were positive). Boxes represents 25th to 75th percentile range, black line demonstrate the median, and whiskers show minimum and maximum values. The horizontal lines represent the medians. *p* values were calculated by Mann–Whitney U test and Kruskal–Wallis test, as appropriate. ns = *p* value > 0.05. (**E**) Spearman correlation between hemoglobin and ferritin levels and serum neutralization titer. ns = *p* value < 0.05.

When SN was employed for further confirmation and assessment of neutralizing capacity of the antibodies produced by vaccines, the results obtained corresponded well with ELISA since 95.24% (60/63) of the study group and 95.52% (64/67) of the control group tested positive (*p* > 0.99, 95% CI = 0.2416 to 4.706). When IgG NP sero-positive samples were excluded, a negligible change was observed, as shown in Figure 4A. Where 94% (47/50) and 95.83% (46/48) of the study group and control, respectively, remained positive expressing no significant difference. We further assessed whether iron deficiency may affect the titer of neutralizing antibodies, and we did not identify any significant difference between the study group and controls that persisted even after anti-NP sero-positive samples were excluded (Figure 4A,B). Similar findings were obtained when the study group was subdivided into ID and IDA (Figure 4C,D). Moreover, we did not find a statistically significant correlation between hemoglobin and ferritin levels with serum neutralization titer (Figure 4E).

**Figure 5:**
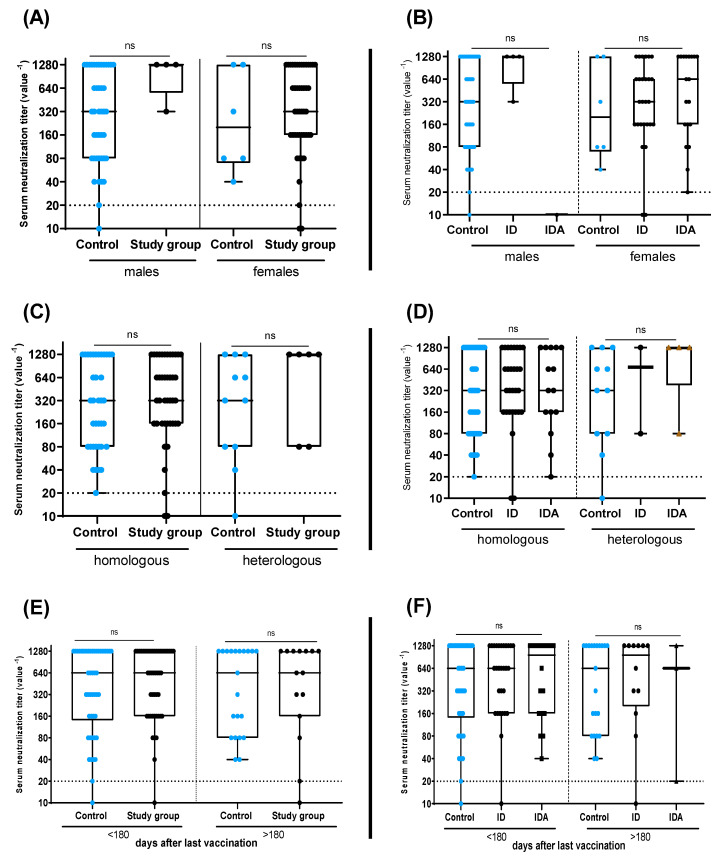
The effect of different variables on the neutralization titer among control group (normal hemoglobin and ferritin) and study group (right panel: ID and IDA combined; left panel: ID and IDA separated). Neutralizing antibody titers related to gender (**A**,**B**), type of vaccine received (**C**,**D**), and period since vaccination (**E**,**F**). Boxes represents 25th to 75th percentile range, black line demonstrate the median, and whiskers show minimum and maximum values. Mann–Whitney U test and Kruskal–Wallis test were used as required. ns indicates non-significant.

Finally, when the scale was adjusted for gender (Figure 5A,B), type of vaccine administered (homologous or heterologous) (Figure 5C,D), or number of days since vaccination (Figure 5E,F), and while excluding anti-NP positive samples, no significant differences were observed. Again, combining or separating ID and IDA in the study group did not affect our findings.

## 4. Discussion

ID is among the most frequent micronutrient insufficiency, impacting billions globally [27]. Iron is required for efficient induction of host immunity following immunization by infections or vaccinations [14,28,29,30]. Iron is known to be necessary for the differentiation of monocytes and macrophages as well as for some antimicrobial activities [10]. Additionally, the generation of cytokines, lymphocyte differentiation, and proliferation require iron [11,12]. Recently, lower hemoglobin and ferritin levels were strongly linked to severe outcomes of COVID-19, which placed a great burden on the vaccines to resolve that problem [16,17,18,19,20]. Yet, lower ferritin and hemoglobin levels have also been suggested to pose challenges for some vaccines effectiveness (e.g., Rubella, DPT, *Haemophilus influenzae* type b (Hib), *Streptococcus pneumoniae* serotype 1 (PS1)) [7,13,14,27]. Although a recommendation to correct iron level before receiving COVID-19 vaccination was raised, the picture with regards to the effect of iron level on COVID-19 vaccines effectiveness is still vague. A study has introduced a ferritin-based COVID-19 vaccine to mice to assess whether ferritin can boost vaccines-induced antibodies. Impressively, their vaccine induced high titers of efficient neutralizing antibodies that lasted for more than seven months compared to a control group that received ferritin lacking equimolar vaccine [31]. To our knowledge, there is a lack of reports about COVID-19 vaccines effectiveness specifically targeting individuals with ID and IDA. Hence, this study to our knowledge is considered the first to remove the fog upon that issue.

In this study, the prevalence of anti-S and anti-NP antibodies were assessed using our lab-developed S and NP based ELISAs and their neutralizing capacity was assessed using SN assay [32,33,34]. Our primary target population was iron deficient individuals and we recruited healthy individual as a control group, from both genders. All study participants have received two doses of COVID-19 vaccines either homologous or heterologous (BNT162b2 by Pfizer-BioNTech, mRNA-1273 by Moderna, and ChAdOx nCov-2019 by AstraZeneca). The S-based ELISA results identified anti-S IgG in nearly all subjects -apart from four- regardless of their ferritin or hemoglobin status. This indicates that the formerly mentioned vaccines have successfully induced anti-S antibodies in both ID and IDA patients and healthy controls. The four subjects who tested negative for anti-S IgG, three of them belonged to the control group and the other one was among the study group (ID and IDA). Two of the three subjects were smokers and the third had asthma, while the one in study group was IDA with no other comorbidities. Harnessing SN to evaluate the vaccines-induced antibodies neutralization capacity was a core milestone in this study. As it is considered the gold standard method to measure antibodies titers and their neutralization activities [35]. SN results showed almost exact resemblance to S-based ELISA results. No significant difference was observed between controls and study group, male and females, and those who received homologous or heterologous vaccines as seen in similar studies [36,37]. Further, we have analyzed the data of ID and IDA separately given the fact that they represent two distinct clinical conditions. Our data remained intact and no significance difference was observed when the study group was separated into ID and IDA groups. This indicates that COVID-19 vaccines are highly effective in inducing the production of highly efficient neutralizing antibodies in humans notwithstanding lower hemoglobin or ferritin levels. Two recent studies conducted on hemodialysis patients identified a positive correlation between hemoglobin and ferritin levels and anti-S antibody titer [38,39]. This may suggest that their finding might have been influenced by other factors. In addition, the presence of anti-SARS-CoV-2 antibodies rather than neutralization activity was considered in these studies [38,39]. However, the findings of our study may not be utterly conclusive when considering the small sample size. Another limitation of this study was the distribution of male and female among control and study groups. Most of the study group were female compared to dominance of male in the control group, which is expected taking into consideration that iron deficiency is more prevalent in females due to several factors (e.g., menstruation, pregnancy, and malnutrition) [40]. Although controversial, others and we have previously shown that gender does not affect COVD-19 vaccine-induced humoral immunity, which suggest that gender suboptimal distribution may have a negligible or no effect on our study findings [41,42,43]. Yet, similar studies with larger sample sizes and more appropriate gender distribution remain necessary to draw comprehensive conclusions about the effect of Iron on COVID-19 vaccine effectiveness. It is not until then that one can advocate for or against the recommendation of correcting the iron level prior to vaccination.

When NP-based ELISA results were analyzed, 53.83% (14/26) of the participants who reported previous COVID-19 infection tested negative for anti-NP IgG regardless to the duration between infection and sample collection. These data suggest that after infection natural immunity may wane which was observed in previous studies [44,45,46,47]. Furthermore, we were able to detect anti-NP IgG in 46.17% (12/26) of participants who reported previous COVID-19 infection even after more than two years from the last infection. These findings propose that the durability of infection-induced antibodies is to some context distinct from person to person. However, upon a second look at other positive NP-based ELISA results, a notable bigger proportion (62.50%, 20/32) of the participants belonged to those who did not report a previous infection; highlighting the participants’ lack of awareness over a possible SARS-CoV-2 infection prior to sample collection [46,47,48,49]. This unawareness of previous infection can be explained, at least in part, by the circulation of rapidly transmitting less virulent variants of SARS-CoV-2, or vaccine-mediated reduction of disease severity.

## 5. Conclusions

Low iron levels have always been implicated in abnormal immune responses. Hence, ID and IDA conveyed concerns for COVID-19-vaccine effectiveness. This study demonstrated that COVID-19 vaccines successfully induced neutralizing-antibody production in individuals with ID and IDA to similar levels observed in healthy controls. Further analyses indicate that the incidence of past infection, type of COVID-19 vaccine, or period since last vaccination did not significantly affect the presence or titer of the vaccine-mediated neutralizing anti-SARS-CoV-2 antibodies. This study was limited by the sample size and gender bias, which necessitates similar studies with larger sample size and more appropriate gender distribution to confirm our findings.

## Figures and Tables

**Table 1:** Characteristics of control (normal hemoglobin and ferritin) and study group (ID, IDA).

Variables		Control (*n* = 67)		Study Group (*n* = 63)		*p* Value
Hemoglobin (g/dl)		15.6 ± 1.74		12.39 ± 1.38		<0.0001
Ferritin (ng/mL)		66.3 ± 29.13		13.3 ± 12.16		<0.0001
Age		21.42 ± 2.48		22.47 ± 6.2		0.854
Body mass index (BMI)		25.54 ± 6.65		23.13 ± 5.29		0.0998
Gender	Male	60	89.55%	5	7.94%	<0.0001
	Female	7	10.45%	58	92.06%	
Previously diagnosed with COVID-19 by RT-PCR	Yes	12	17.91%	14	22.22%	0.6617
	No	55	82.09%	49	77.78%	
Vaccination	Homologous	53	79.10%	55	87.30%	0.2477
	Pfizer	48	71.64%	53	84.13%	
	AstraZeneca	5	7.46%	2	3.17%	
	Heterologous	14	20.90%	8	12.70%	
	Pfizer/AstraZeneca	14	20.90%	7	11.11%	
	AstraZeneca/Moderna	0	0.00%	1	1.59%	
Days since vaccination	<180	46	68.66%	48	76.19%	0.4332
	>180	21	31.34%	15	23.81%	

## Data Availability

Data sharing not applicable.
